# Effects of Acute Lithium Treatment on Brain Levels of Inflammatory Mediators in Poststroke Rats

**DOI:** 10.1155/2015/916234

**Published:** 2015-09-27

**Authors:** Matthew Boyko, Ahmad Nassar, Jacob Kaplanski, Alexander Zlotnik, Yael Sharon-Granit, Abed N. Azab

**Affiliations:** ^1^Department of Anesthesiology and Critical Care, Soroka University Medical Center, Faculty of Health Sciences, Ben-Gurion University of the Negev, P.O. Box 653, 84105 Beer-Sheva, Israel; ^2^Department of Clinical Biochemistry and Pharmacology, Faculty of Health Sciences, Ben-Gurion University of the Negev, P.O. Box 653, 84105 Beer-Sheva, Israel; ^3^School for Community Health Professions, Faculty of Health Sciences, Ben-Gurion University of the Negev, P.O. Box 653, 84105 Beer-Sheva, Israel

## Abstract

Stroke is a leading cause of mortality and morbidity worldwide. Few therapeutic options with proven efficacy are available for the treatment of this disabling disease. Lithium is the gold standard treatment for bipolar disorder. Moreover, lithium has been shown to exhibit neuroprotective effects and therapeutic efficacy as a treatment of other neurological disorders. This study was undertaken to examine the effects of lithium on brain inflammatory mediators levels, fever, and mortality in postischemic stroke rats. Ischemic stroke was induced by occlusion of the mid cerebral artery (MCAO). Pretreatment with a single dose of lithium at 2 hours before MCAO induction significantly reduced the elevation in interleukin- (IL-) 6 and prostaglandin E_2_ levels in brain of post-MCAO rats, as compared to vehicle-treated animals. On the other hand, lithium did not affect the elevation in IL-1*α*, IL-10, IL-12, and tumor necrosis factor-*α* levels in brain of post-MCAO rats. Moreover, pretreatment with lithium did not alter post-MCAO fever and mortality. These results suggest that acute pretreatment with a single dose of lithium did not markedly affect post-MCAO morbidity and mortality in rats.

## 1. Introduction

In 2004 the World Health Organization reported that stroke was the second cause of death worldwide after ischemic heart disease [1]. In the United States, stroke was ranked as the third cause of death after cardiovascular diseases and cancer [2]. Moreover, cerebrovascular disease was ranked as the first or second leading cause of burden of disease in the Western Pacific and European regions, respectively [1]. Those data clearly indicate that stroke is a very fatal and disabling disease.

The Atherosclerosis Risk in Communities study has shown that* ischemic* stroke was the most prevalent (83%) type of stroke among a cohort of 15792 Americans aged 45–64 years, followed by intracerebral (10%) and subarachnoid (7%) hemorrhages [3]. The mechanisms underlying the pathological processes that take place in the brain after the occurrence of ischemic stroke are very complex. Cerebral ischemia (due to decreased blood flow to brain tissue) activates an ischemic cascade which leads to cell death and severe neuronal damage. A large body of evidence suggested that inflammation plays a pivotal role in the pathological processes in poststroke brain [4]. Postischemic inflammation involves activation of glial cells which produce cytotoxic and cytoprotective mediators [4]. After the occurrence of the ischemic injury, activated glial cells produce and secrete inflammatory mediators such as interleukin- (IL-) 1*α*, IL-1*β*, IL-6, IL-10, IL-12, nitric oxide, prostaglandin E_2_ (PGE_2_), and tumor necrosis factor- (TNF-) *α* [4–7]. Moreover, peripherally produced cytokines and inflammatory mediators produced by brain infiltrated immune cells also contribute to the postischemic inflammatory response [4]. Despite the data suggesting that poststroke inflammation harms infarct zone tissue, it is worth noting that some immune/inflammatory processes enhance brain recovery and attenuate neuronal damage in postinsult brain [4]. Thus, inhibition of inflammation does not always benefit the brain.

Many poststroke patients present with changes in body temperature (BT), mostly hyperthermia (occasionally referred to as fever or pyrexia) [8, 9]. Poststroke fever has been associated with a prominent increase in mortality, morbidity, and hospital length of stay [8, 9]. For example, Castillo et al. [8] found that mortality rate was significantly higher among hyperthermic as compared to normothermic poststroke patients at 3 months (15.8% versus 1%, resp., *P* < 0.001). In addition, they observed that hyperthermia during the first 24 hours after stroke onset was independently and significantly associated with larger infarct volume, higher neurological deficit, and dependency at 3 months after stroke [8].

The pharmacotherapy of stroke includes few agents with proven efficacy [10] underscoring the need for novel efficacious treatments. Lithium is a monovalent cation widely and effectively used in the treatment of bipolar affective disorder [11]. Moreover, lithium has been shown to confer potent neuroprotective effects in various experimental models [12, 13]. Lithium also exerts multiple effects on immune and inflammatory processes in the brain including some potent anti-inflammatory properties [14]. As mentioned, inflammation contributes to the neuronal damage which occurs in poststroke brain. Therefore, the primary objective of this study was to examine the effects of acute lithium treatment on levels of inflammatory mediators in brain of poststroke rats. In addition, we aimed to determine the effects of lithium on poststroke fever and mortality.

## 2. Materials and Methods

### 2.1. Animals

Male Wistar rats were used throughout the studies (Table 1). The animals were housed 3 per cage and maintained under controlled environmental conditions (ambient temperature 22 ± 1°C, relative humidity 55–58%, and photoperiod cycle 12 h light : 12 h dark), fed Purina Lab Chow and water* ad libitum*. Only animals with no overt pathology were included in the studies. The procedures of the study were in accordance with the Guidelines of the Committee for the Use and Care of Laboratory Animals in Ben-Gurion University of the Negev, Israel.

### 2.2. Lithium Treatment

Lithium chloride (LiCl, purchased from Sigma) was dissolved in NaCl 0.9% and filtered to produce a sterile solution. Rats were treated with a single intraperitoneal (ip) injection of LiCl 100 mg/kg at 2 h before induction of anesthesia. Alternatively, control animals were treated with vehicle (0.35 mL sterile NaCl 0.9%, ip).

### 2.3. Surgical Procedure

Permanent mid cerebral artery occlusion (MCAO) was performed according to the method of Longa et al. [15] with slight modifications as described previously [16]. Briefly, rats were anesthetized with ketamine 75 mg/kg and midazolam 3 mg/kg (both given ip). Anesthetized rats were subjected to the surgical procedure which lasted 25–30 min during which they were allowed to breathe spontaneously. The right common carotid artery (CCA) was exposed through a middle neck incision and carefully dissected from surrounding tissues from its bifurcation to the base of the skull. The occipital artery branches of the external carotid artery (ECA) were then isolated, dissected, and coagulated. The ECA was further dissected distally and coagulated along with the terminal lingual and maxillary artery branches. The internal carotid artery (ICA) was isolated and carefully separated from the adjacent vagus nerve and the pterygopalatine artery was ligated close to its origin with a 4-0 silk suture. Then, the MCA was blocked by inserting a 3.5 cm length of a 4-0 nylon silicon-coated filament 18–18.5 mm before the bifurcation of the CCA and then into the circle of Willis, effectively occluding the MCA (a procedure hereafter referred to as MCAO). The ICA and CCA were temporarily blocked. Subsequently, a 4-0 silk suture was tied loosely around the CCA before its bifurcation. The silk suture around the CCA stump was fastened around the intraluminal filament to prevent bleeding. The filament was then fixed by tying up a silk suture over the CCA. The suture was left in place permanently. Sham-operated (control) rats were anesthetized and subjected only to a middle neck skin incision.

### 2.4. Measurement of Body Temperature

BT was measured with a plastic-coated thermocouple probe (HL 600 Thermometer, Anristu Meter Co., Japan) inserted into the rectum. Measurement was performed at 2 h after surgery in both sham- and MCAO-operated rats.

### 2.5. Assessment of Postsurgical Neurological Deficit and Mortality

In order to verify the correctness of the MCAO procedure animals were tested for existence of a neurological deficit (ND) [16]. Two observers who were blind to type of the surgical procedure performed examined each animal for visible NDs. Any of the following features was regarded as a ND: forelimb flexion; contralateral forelimb gripping weakly (the operator placed the rat on an absorbent pad and gently pulled the tail); circling to the paretic side only when pulled by the tail (the rat was allowed to move freely on the absorbent pad); spontaneous circling. Rats in the MCAO group needed to present at least one of the ND features above in order to be included in this group (Table 1). Rats in the sham group needed to present no visible NDs in order to be included in this group (were excluded if they presented any visible ND). Moreover, we assessed mortality during 24 h following the surgical procedure. The rate of mortality was compared between rats of the* same* surgical procedure; namely, sham + vehicle group was compared to sham + LiCl group and MCAO + vehicle group was compared to MCAO + LiCl group (Table 1).

### 2.6. Preparation of Brain Homogenates

At 24 h after the MCAO procedure surviving rats were anesthetized briefly (with a mixture of 4% isoflurane in 100% oxygen) and immediately sacrificed by decapitation. Brains were quickly extracted and washed in ice-cold saline 0.9%. Frontal cortex (FC), hypothalamus (HT), and hippocampus (HC) were gently excised on ice, cleaned, and immediately transferred to –80°C. Then, each sample was weighed and manually homogenized for 10 seconds in 500 *μ*L of a cold phosphate-buffered saline solution containing protease inhibitors (homogenizing buffer). Subsequently, tissue homogenates were centrifuged at 10,000 rpm, 4°C for 10 min. Supernatants were collected and immediately transferred to –80°C for further determination.

### 2.7. Determination of PGE_2_ and Cytokines Levels

Samples (supernatants) were assayed for PGE_2_ protein content using an ELISA kit according to manufacturer's instructions (R&D Systems; Minneapolis, MN, USA). Levels of IL-1*α*, IL-6, IL-10, IL-12, and TNF-*α* were determined by a multiplexed ELISA array (Quansys Biosciences; Logan, Utah, USA). This assay allows measuring the concentration of multiple inflammatory-associated mediators in a single sample. In both assays, when the level of the detected mediator was below the lower detection limit, results were annotated as “below detection limit” and assigned a result of zero.

### 2.8. Statistical Analysis and Presentation of the Data

We performed two independent experiments. The number of rats in each group in the* first* experiment,* second* experiment, and* total* count was as follows, respectively: sham + vehicle—3, 6, and 9; sham + LiCl—3, 6, and 9; MCAO + vehicle—12, 17, and 29; MCAO + LiCl—11, 17, and 28. Statistical evaluations were carried out using Student's* t*-test (two-tailed) or chi-square Fisher exact test, according to type of the tested parameter. Normally distributed data and continuous variables are presented as mean ± SEM. Values of *P* < 0.05 were considered statistically significant. It is worth noting that all brain samples were homogenized in 500 *μ*L of homogenizing buffer regardless of their weight. Thus, the content of inflammatory mediators in resultant homogenates was lower than their actual content in the original tissue (taking into account that, e.g., the average weight of HT samples was 41 mg while the average weight of FC samples was 45 mg). Results in figures of IL-1*α*, IL-6, IL-10, IL-12, PGE_2_, and TNF-*α* were calculated as follows: ELISA result (pg/mL) divided by sample weight in milligrams. Results are presented as pg/mL (pg/mg wet weight).

## 3. Results

### 3.1. Effects of Acute Lithium Treatment on Body Temperature of Poststroke Rats

**Figure 1:**
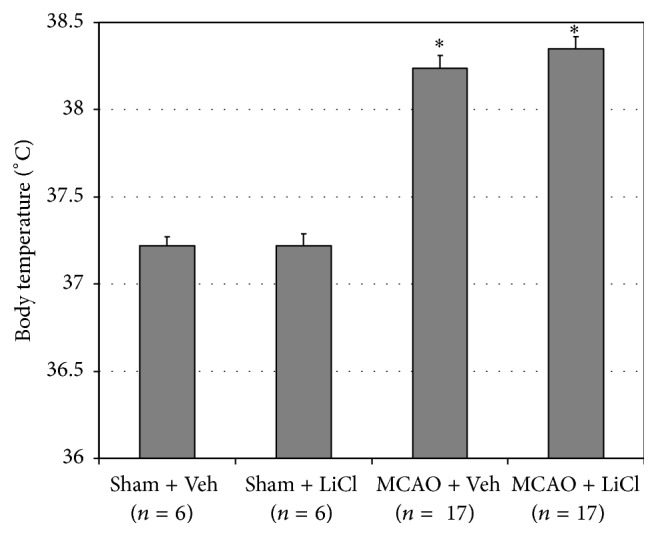
Effects of lithium on body temperature of poststroke rats. At 2 h before surgery vehicle-treated rats were injected (ip) with 0.35 mL NaCl 0.9% and LiCl-treated rats with 100 mg/kg lithium. BT was measured at 2 h after surgery as described in Section 2. This figure represents the results of the* second* experiment of the study. The results in the first experiment were similar to those presented in this figure. Each column is the mean ± SEM of 6 or 17 rats per group as indicated in the figure. ^*∗*^ *P* < 0.05 versus sham + Veh. LiCl, lithium chloride; MCAO, middle cerebral artery occlusion; Veh, vehicle.

Before surgery, BT did not significantly differ between all treatment groups (data not shown). As compared to sham-operated rats, MCAO-operated rats had a significantly higher BT at 2 h after surgery (Figure 1). Pretreatment with lithium did not alter BT either in sham-operated or in MCAO-operated rats (Figure 1).

### 3.2. Effects of Acute Lithium Treatment on Mortality of Poststroke Rats

At 24 h after surgery all sham-operated rats survived and exhibited no visible NDs (Table 1). On the other hand, all rats that underwent MCAO surgery and survived had visible NDs (Table 1). Mortality was followed during 24 h* after* surgery. Animals that died during the surgical procedure were not counted as part of the MCAO groups. Out of 60 rats that underwent MCAO surgery in the two experiments of the study, 3 died* during* the surgical procedure (5% perioperative mortality). Thus, mortality was assessed in the remaining 57 MCAO rats. Eight out of 29 vehicle-treated MCAO rats died during 24 h after surgery (27.6% mortality, Table 1). Seven out of 28 lithium-treated MCAO rats died during 24 h after surgery (25% mortality, Table 1). These results indicate that acute pretreatment with lithium did not significantly (*P* = 0.532) reduce the rate of poststroke mortality.

### 3.3. Effects of Acute Lithium Treatment on Brain Inflammatory Mediators Levels of Poststroke Rats

**Figure 2:**
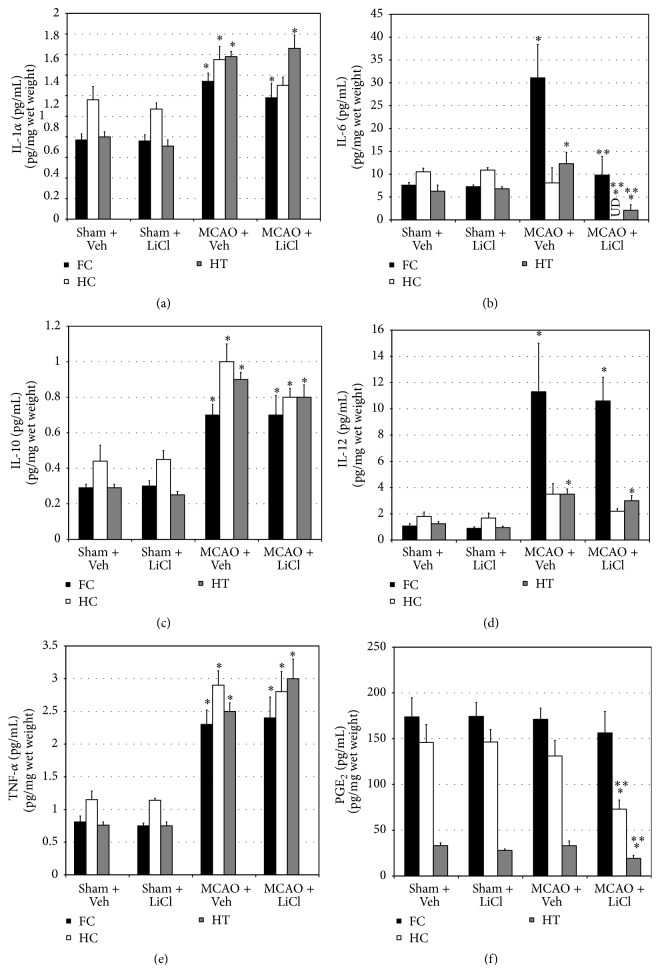
Effects of lithium on brain inflammatory mediators levels of poststroke rats. At 2 h before surgery vehicle-treated rats were injected (ip) with 0.35 mL NaCl 0.9% and LiCl-treated rats with 100 mg/kg LiCl. At 24 h after surgery* surviving* rats were sacrificed and their brains were quickly excised. Then, FC, HC, and HT were excised and stored in −80°C. Levels of IL-1*α* (a), IL-6 (b), IL-10 (c), IL-12 (d), TNF-*α* (e), and PGE_2_ (f) in brain regions were measured as described in Section 2. This figure represents the results of the* second* experiment of the study. The results in the first experiment were similar to those presented in this figure. Each column is the mean ± SEM of 6 (sham + Veh and sham + LiCl) or 12 (MCAO + Veh and MCAO + LiCl) rats per group. ^*∗*^ *P* < 0.05 versus sham + Veh; ^*∗∗*^ *P* < 0.05 versus MCAO + Veh. FC, frontal cortex; HC, hippocampus; HT, hypothalamus; LiCl, lithium chloride; MCAO, middle cerebral artery occlusion; UD, undetectable; Veh, vehicle.

As compared to sham-operated rats, levels of IL-1*α*, IL-6 (except in HT), IL-10, IL-12 (except in HT), and TNF-*α* were significantly higher in brain regions of MCAO-operated rats (Figures 2(a), 2(b), 2(c), 2(d), and 2(e), resp.). Pretreatment with lithium did not alter the levels of IL-1*α*, IL-10, IL-12, and TNF-*α* either in sham-operated or in MCAO-operated rats (Figures 2(a), 2(c), 2(d), and 2(e)). On the other hand, pretreatment with lithium significantly reduced IL-6 levels in all brain regions in MCAO-operated rats (Figure 2(b)). Moreover, PGE_2_ levels were similar in brain regions of sham- and MCAO-operated rats (Figure 2(f)). Surprisingly, pretreatment with lithium significantly reduced PGE_2_ levels in HC and HT of MCAO-operated but not in sham-operated rats (Figure 2(f)).

## 4. Discussion

The present study demonstrated that acute pretreatment with lithium did not prominently alter brain inflammation in post-MCAO rats. Lithium significantly reduced levels of IL-6 in FC, HC, and HT and PGE_2_ in HC and HT in post-MCAO rats. However, it did not influence the elevation in IL-1*α*, IL-10, IL-12, and TNF-*α* levels in post-MCAO rats. In addition, under the experimental conditions used in this study, pretreatment with lithium did not alter post-MCAO fever and mortality.

Lithium is the gold standard pharmacotherapy of bipolar disorder [12]. Lithium was also shown to exert therapeutic benefits in the treatment of other neurological disorders including Alzheimer's disease [17] and amyotrophic lateral sclerosis [18]. The pathophysiology of stroke comprises a prominent inflammatory response, particularly in the postischemic zone. The ischemic cascade leads to dysregulation of brain function and homeostasis due a profound oxidative stress and prominent subsequent inflammation, both of which lead to cell death and aggravation of tissue damage [4]. Moreover, lithium was found to exert anti-inflammatory [14] and neuroprotective [12, 13] effects under various experimental conditions. Therefore, we hypothesized that lithium may possess anti-inflammatory effects in poststroke rats. Under the experimental conditions used in the present study lithium reduced the levels of IL-6 and PGE_2_ but did not affect the levels of IL-1*α*, IL-10, and IL-12 in poststroke rats (Figure 2).

PGE_2_ is an important mediator of tissue homeostasis and alteration of its level/function may lead to deleterious pathological processes [19]. A large body of evidence associated PGE_2_ with inflammatory conditions and pathological changes in BT. For example, PGE_2_ was shown to contribute to the pathogenesis of lipopolysaccharide- (LPS-) induced hypothermia and fever in rats [20]. Nonsteroidal anti-inflammatory drugs (NSAIDs) such as aspirin and ibuprofen inhibit the production of PGE_2_ and are widely used as antipyretic medications [19]. Chronic lithium treatment was found to reduce brain levels of PGE_2_ in rats [21]. Moreover, we showed that acute pretreatment with lithium significantly decreased hypothalamic PGE_2_ levels in LPS-treated rats, which was accompanied by a significant attenuation of LPS-induced hypothermia [20]. Importantly, permanent MCAO was associated with a significant hyperthermia and increased hypothalamic damage in rats [22]. In the present study we examined the effect of acute pretreatment with lithium on poststroke fever. We found that MCAO-operated animals had a significantly higher BT than sham-operated rats at 2 h after surgery (Figure 1). Lithium did not influence the fever in MCAO-operated rats (Figure 1), despite a significant reduction in hypothalamic PGE_2_ levels (Figure 2). Surprisingly, HT PGE_2_ levels did not differ between MCAO-operated and sham-operated animals (Figure 2), despite the reported damage to the hypothalamus in post-MCAO-operated rats [22]. These findings hint at a possibility that PGE_2_ does not play a central role in the mechanism of poststroke fever. Consistent with this assumption, Legos et al. [23] found that aspirin, a NSAID which inhibits PGE_2_ production, did not attenuate hyperthermia in MCAO-operated rats at 2 h after surgery. It is reasonable to assume that poststroke fever results (at least in part) from and is associated with the tissue damage in the ischemic zone. The dissemination of cell debris and burst of excitatory neurotransmitters (such as glutamate) secretion contribute to the development of poststroke fever [24], which does not necessarily respond to systemic administration of NSAIDs [24].

IL-1*α* is usually regarded as a* proinflammatory* cytokine [25]. Under resting conditions IL-1*α* constitutively presents at low levels in multiple cell types; however, following tissue damage, cell death, or hypoxia, its expression is greatly induced and it is produced by a number of cells such as macrophages, neutrophils, and epithelial cells [25]. IL-1*α* facilitates the recruitment of immune cells and induces the secretion of mainly proinflammatory cytokines such as IL-1*β* and TNF-*α* [25]. Several studies have associated IL-1*α* with the pathophysiology of ischemic stroke [26]. For example, Luheshi et al. [26] reported that IL-1*α* production is upregulated early after the occurrence of ischemic stroke in mice, leading to induction of proinflammatory cytokines secretion and aggravation of inflammation in the ischemic-injured zone. In the present study we found that IL-1*α* levels were significantly increased in FC, HC, and HT of MCAO-operated rats (Figure 2). Pretreatment with lithium did not affect the elevation in IL-1*α*.

IL-6 acts mostly as a* proinflammatory* cytokine but it also exerts some anti-inflammatory properties [27]. It is produced by T cells, macrophages, and other cells in response to immune activation due to infection or tissue damage. IL-6 has been associated with pathological conditions such as arthritis, autoimmune and inflammatory responses, and cancer [27]. IL-6 is also involved in the regulation of metabolic, regenerative, and neuronal processes [27]. Elevated plasma and CSF levels of IL-6 were associated with increased neurological worsening in patients with ischemic stroke [11]. In the present study, IL-6 levels were significantly increased in FC and HT of MCAO-operated rats. Pretreatment with lithium significantly reduced IL-6 levels in FC and HT (Figure 2), pointing to a possible anti-inflammatory effect of the drug in these brain regions. Surprisingly, lithium did not alter IL-6 levels in HC of sham-operated rats but totally abolished its levels in MCAO-operated rats. The reason for this discrepancy is currently not understood.

IL-10 is an* anti*-*inflammatory* cytokine which inhibits the activity of proinflammatory cytokines and suppresses the expression of their receptors [28]. It is secreted under different conditions of immune activation by a variety of cells including T and B cells, monocytes, macrophages, and glial cells. IL-10 suppresses inflammatory responses and plays a role in maintaining homeostasis of overall immune responses, including during conditions of neuroinflammation [28]. Tarkowski et al. [5] found that IL-10 levels were increased in CSF of stroke patients. Decreased plasma levels of IL-10 were associated with increased neurological worsening in patients with ischemic stroke [29]. Moreover, IL-10-producing T cells were found to play a role in reducing infarct size volume in post-MCAO mice [7]. In the present study, IL-10 levels were significantly increased in FC, HC, and HT of MCAO-operated rats (Figure 2). Pretreatment with lithium did not alter IL-10 levels in those brain regions.

The IL-12 family of cytokines comprises a number of members including IL-12, IL-23, IL-27, and IL-35 [30]. IL-12 is composed of two subunits, p35 and p40, which bind to and activate IL-12 receptors. IL-23 and IL-35 are also composed of 2 subunits (heterodimers) in which p40 and p35, respectively, also activate IL-12 receptors [30]. The regulation of the inflammatory response by these cytokines is influenced by the identity of the subunits composing the cytokine heterodimer. For example, IL-23 and IL-12 are proinflammatory cytokines while IL-35 is an anti-inflammatory cytokine. A number of studies addressed the association between IL-12 and ischemic stroke. For example, Konoeda et al. [31] found that treatment with an anti-p40 monoclonal antibody (which blocks the activity of IL-12 and IL-23) decreased ischemia/reperfusion injury and enhanced recovery of neurological deficits in mice. Moreover, Narasimhalu et al. [32] reported that increased serum level of IL-12 was associated with a cognitive decline in postischemic stroke patients. In the present study IL-12 levels were significantly increased in FC and HT of MCAO-operated rats, which was not affected by pretreatment with lithium (Figure 2).

TNF-*α* is an important multifunctional* proinflammatory* cytokine secreted from various immune and glial cells [33]. It stimulates numerous immune/inflammatory responses and regulates various physiological as well as pathological processes in humans. TNF-*α* has been associated with the pathophysiology of many neurological illnesses including psychiatric and neurodegenerative disorders [5, 33, 34]. For example, a comprehensive meta-analysis of 30 studies has demonstrated that plasma levels of TNF-*α* are increased in bipolar patients as compared to control subjects [34]. Importantly, it was found that TNF-*α* levels are increased in CSF of stroke patients [5]. Lithium was shown to decrease TNF-*α* levels under multiple experimental conditions; however, several contradicting findings have also been reported (reviewed in [14]). In the present study TNF-*α* levels were significantly increased in FC, HC, and HT of MCAO-operated rats, which was not affected by pretreatment with lithium (Figure 2).

Relying on evidence attesting for the involvement of inflammation in the pathophysiological mechanisms underlying ischemic stroke, several studies have examined the therapeutic potential of different anti-inflammatory strategies as a treatment for ischemic stroke. For example, Caso et al. [35] examined the effect of deleting (knocking out) toll-like receptor- (TLR-) 4 in post-MCAO mice. As compared to their respective controls, TLR4-deficient mice had a diminished brain inflammatory response which was accompanied by a significant decrease in infarct volume and neurological and behavioral alterations [35]. Similarly, Zhou et al. [36] reported that MCAO resulted in a significant cerebral infarction and increased neurological deficit scores in rats. Treatment with propofol (an anesthetic drug which confers anti-inflammatory properties) significantly reduced infarct volume and improved neurological function in MCAO-operated rats [36].

A number of experimental studies have investigated the utility of lithium as a treatment for ischemic stroke suggesting that it has potent therapeutic benefits. For example, Nonaka and Chuang [37] found that chronic treatment with lithium (subcutaneously for 16 days) significantly reduced infarct volume and neurological deficits in post-MCAO rats. Similarly, Xu et al. [13] demonstrated that chronic subcutaneous treatment with lithium for 16 days significantly decreased infarct volume and neurological deficits in post-MCAO rats. Moreover, Sheng et al. [38] reported that a single intravenous injection with lithium led to a significant reduction in infarct volume and neurological deficits in post-MCAO rats. Interestingly, this study showed that a combinatory treatment with lithium + PGE_1_ resulted in greater neuroprotection than treatment with each agent alone [38]. Despite the encouraging results obtained in the cited animal studies, a randomized, placebo-controlled, double-blind clinical trial in stroke patients revealed that treatment with lithium for 30 days did not significantly improve patients' motor recovery [39]. Patients were randomly assigned to receive lithium (*n* = 32) or placebo (*n* = 34) during the first 48 hours after stroke onset and treatment was continued for 30 days. Improvement measures did not differ significantly between the groups. However, in a subgroup of patients with cortical stroke lithium resulted in a significant improvement in motor activity. The authors suggested that cortical stroke patients may particularly benefit from lithium treatment and advocated conduction of a larger sample size trial to test this assumption [39]. In the present study lithium treatment did not decrease NDs and mortality in MCAO-operated rats. The reason for the discrepancy between the results (positive) of previous animals studies [13, 37, 38] and those of the present study is not clear. A possible reason could be that our study utilized an acute lithium treatment regimen (single dose, ip) while most of the previous studies used a chronic treatment protocol. We speculated that administering lithium acutely at 2 h before induction of MCAO would be sufficient to inhibit the major enzymes that are regarded as therapeutic targets of the drug (e.g., inositol monophosphatase and glycogen synthase kinase-3). Indeed, treatment with lithium* in vitro* inhibits inositol monophosphatase and glycogen synthase kinase-3 within minutes after exposure to the drug. Nevertheless, it is still possible that a chronic lithium treatment produces a prominent and long-lasting cellular impact which results in a more profound protective effect against the deleterious results of the ischemic damage.

Our study had some limitations. One limitation was that we measured BT only at 2 h after MCAO. The main reason for this was that mortality occurred as early as 4 hours after (MCAO) surgery. Another limitation was that we administered lithium prophylactically* before* MCAO surgery. Although stroke has several known risk factors, in clinical reality, the exact time for the occurrence of stroke is largely unpredictable. Thus, it could be argued that a more appropriate experimental design would be to examine the efficacy of lithium treatment* after* the occurrence of stroke. Nevertheless, we believe that examining the prophylactic use of lithium (or another drug) as a possible treatment against stroke is worth testing and has scientific merit.

In summary, the present study shows that lithium exhibited some anti-inflammatory properties but did not affect hyperthermia, neurological deficits, and mortality in poststroke rats.

## Figures and Tables

**Table 1:** Effects of lithium on mortality of poststroke rats.

Group	Sham + Veh	Sham + LiCl	*P* value^*∗*^	MCAO + Veh	MCAO + LiCl	*P* value^*∗*^
*n*	9	9	NS	29	28	NS
Weight (g); mean ± SEM	363.4 ± 5.6	362.3 ± 4.7	NS	376.2 ± 3.9	378.3 ± 3.5	0.693
Rats with visible NDs at 24 h; number (%)	0 (0)	0 (0)	NS	21 (100)	21 (100)	NS
Mortality after 24 h; number (%)	0 (0)	0 (0)	NS	8 (27.6)	7 (25)	0.532

At 2 h before surgery vehicle-treated rats were injected (ip) with 0.35 mL NaCl 0.9% and LiCl-treated rats with 100 mg/kg lithium. Existence of NDs was assessed in surviving rats at 24 h after surgery as described in Section 2. Mortality was followed during 24 h after surgery. This table represents the results of the two experiments conducted in this study. ^*∗*^Comparisons for statistical significance were done only between groups that underwent the same surgical procedure, that is, Sham + Veh versus Sham + LiCl; MCAO + Veh versus MCAO + LiCl. LiCl, lithium chloride; MCAO, middle cerebral artery occlusion; ND, neurological deficit; NS, nonsignificant; Veh, vehicle.
